# A Case of Recurrent Transient Monocular Visual Loss after Receiving Sildenafil

**DOI:** 10.1155/2011/645089

**Published:** 2011-12-22

**Authors:** Asaad Ghanem Ghanem

**Affiliations:** Ophthalmology Center, Faculty of Medicine, Mansoura University, Mansoura 35516, Egypt

## Abstract

A 53-year-old man was attended to the Clinic Ophthalmic Center, Mansoura University, Egypt, with recurrent transient monocular visual loss after receiving sildenafil citrate (Viagra) for erectile dysfunction. Examination for possible risk factors revealed mild hypercholesterolemia. Family history showed that his father had suffered from bilateral nonarteritic anterior ischemic optic neuropathy (NAION). Physicians might look for arteriosclerotic risk factors and family history of NAION among predisposing risk factors before prescribing sildenafil erectile dysfunction drugs.

## 1. Introduction

Sildenafil citrate (Viagra; Pfizer Pharmaceuticals, New York, NY, USA) is a selective phosphodiesterase 5 inhibitor and partial phosphodiesterase 6 inhibitor prescribed for erectile dysfunction. Sildenafil intake leads to smooth muscle relaxation in the corpus cavernosum, allowing inflow of blood by enhancing the effect of nitric oxide and cyclic guanosine monophosphatase pathway, during sexual intercourse. Sildenafil is rapidly absorbed with a half-time of four hours with a maximum plasma level reached within half up to two hours after oral intake [1].

Painless transient monocular visual loss is consistent with an ischemia occurring repeatedly in the visual pathway anterior to the chiasm. Other conditions such as intermittent angle-closure glaucoma, pigment dispersion glaucoma, optic disc drusen, and papilloedema can cause monocular blindness. Repeated transient monocular visual loss during sexual intercourse has been reported in relation to subacute angle closure [2], and to hypothetical retinal vasospasm [3]. This case revealed transient monocular visual loss in male patient with hypercholesterolemia, and family history of NAION with the use sildenafil citrate (Viagra) for erectle dysfunction.

## 2. Case Report

A nonsmoker 53-year-old man used sildenafil citrate (Viagra) for erectile dysfunction. History recording tells that he had been using this drug for the last four months at least once a week. The patient had no past medical history related to cardiovascular or erectile disorders and did not take any other treatment. The patients had no history of alcohol intake.

The patient complained of transient painless blurred vision in his left eye, recurring after sexual intercourse, two or three times. Each attack of transient monocular blindness lasted 2-3 minutes, then followed by spontaneous visual recovery. The vision was not disturbed in the fellow eye. Ophthalmic examination revealed a corrected visual acuity of 10/10 for the right eye and 2/10 for the left eye. Pupils reaction revealed a relative afferent pupillary defect of the left pupil. Slit-lamp biomicroscopy of the anterior segments of both eyes was normal, including angles, and did not show any pathological manifestations. Intraocular pressure (IOP) was 16 mmHg for both eyes. The color vision score for the affected left eye was 9 out of 15 Ishihara plates, while the unaffected right eye had a score of 15 out of 15. General and neurological assessments revealed no abnormal findings. Cardiac examination was normal, without arrhythmia or any sources of emboli.

**Figure 1:**
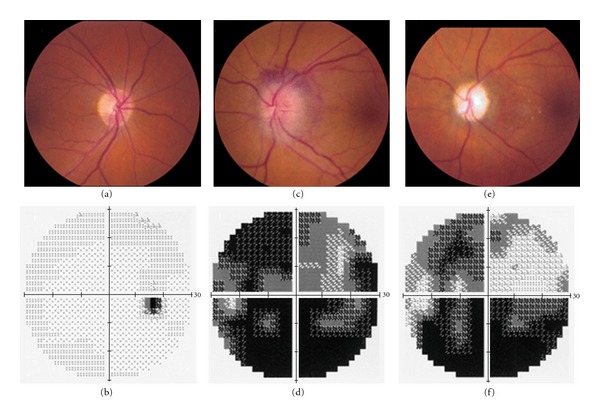
Nonarteritic anterior ischemic optic neuropathy (NAION) in the left eye of a 53-year-old man patient with a history of hypercholesterolemia. The right optic disk is pink and flat (a). Automated perimetry, using the Humphrey visual field 24-2 protocol, shows a normal visual field (b), with the dark spot in the temporal field being the physiologic blind spot. Acutely, the left optic disk appears swollen and hyperemic, with hemorrhage at the superior and temporal disk margin (c). Automated perimetry shows diffuse visual-field loss, more marked in the inferior aspect of the field (d). On review at 6 months, the optic disk swelling has resolved and the disk appears diffusely pale and atrophic (e). Automated perimetry shows improvement in the superior visual field, with a persistent inferior altitudinal defect (f).

Fundus examination revealed swelling and hyperemia of the left optic disc with hemorrhage at superior and inferior disc margins while the vessels, macula, and the peripheral retina were normal (Figure 1). Humphrey visual field (24-2 program) perimetry showed diffuse visual-field loss, more marked in the inferior aspect of the field of the left eye (Figure 1). Fundus fluorescein angiography revealed hyperfluorescence of the left optic disk and leakage from it, indicating edema. There was no intraocular inflammation or pathological disorders. The patient was not hyperopic, and the cup-to-disk ratio in the fellow eye was 0.3.

Laboratory tests excluded diabetes, syphilis, and hypercoagulable states. Antinuclear antibodies and anticardiolipin antibody tests were all negative. In addition, routine blood tests, erythrocyte sedimentation rate, and C-reactive protein were in the normal range. There was, however, mild dyslipidaemia (total cholesterol 248 mg/dL; LDL 156 mg/dL; HDL 52 mg/dL). Chest radiography was normal. A magnetic resonance image scan of the brain and orbits with gadolinium demonstrated normal optic nerves and no white matter lesions. A Doppler ultrasound of the vertebral basilar arteries, the external carotid arteries, and the common carotid arteries did not reveal any significant disorders as stenosis or plaque. Neurological examinations were also normal.

The above-mentioned results led to the conclusion that the patient had experienced a NAION attack on his left eye. He was consulted to discontinue the use of sildenafil citrate. The patient was subjected to two subtenon injections of betamethasone with a three-week interval in between.

Six months after the initial attack, visual acuity improved to 8/10 for the left eye, the optic disk swelling has resolved, and the disk appears diffusely pale, and atrophic automated perimetry shows improvement in the superior visual field, with a persistent inferior altitudinal defect (Figure 1).

## 3. Discussion

NAION is the most common acute optic neuropathy in older age groups with an estimated annual incidence of 2.3 to 10.2 per 100,000 over 50 years of age, and 0.54 per 100,000 for all age [4, 5]. It is presumed to result from vascular insufficiency at the optic nerve head leading to ischemia, but the specific mechanism of the vasculopathy remains unproven. The first visual symptoms noticed by patients with NAION are typically blurred vision, loss of part of the visual field, or both. A diffuse or focally swollen optic disc is observed and multiple flame-shaped hemorrhages are usually present.

Hypovolemia, mainly due to severe surgical procedures [6], atherosclerotic risk factors, [7], hypercoagulable states [8],and crowded optic disk [9], in possible combination with regional vascular endothelial disorders has been reported in the pathophysiology of NAION.

The role of sildenafil in causing injury to the optic nerve is not known. Sildenafil citrate is a selective phosphodiesterase 5 inhibitor causing vasomotor effects through its action on the nitric oxide-cyclic GMP pathway. Nitric oxide is a potent vasodilator, and it physiologically regulates the blood pressure. Inhibition of nitric oxide synthetase in an animal model for glaucoma rescued retinal ganglion cell from damage [10]. Although nitric oxide may play a role in IOP regulation, sildenafil did not cause an increase in IOP in healthy male volunteers [11].

Several studies reported that there has been an increasing number of case reports concerning patients who have developed NAION soon after the use of sildenafil and other phosphodiesterase type 5 inhibitors [12, 13]. It has been hypothesized that these agents might exaggerate the physiologic nocturnal hypotension resulting in ischemia to the optic nerve head or that they might interfere with the autoregulation of blood flow thereby decreasing perfusion to the optic nerve head [14].

In most of the cases of NAION after the use of sildenafil, patients detected visual loss upon awakening in the morning. Elevated levels of sildenafil and its active metabolite are present in the blood for 8 to 12 hours after ingestion. Therefore, if sildenafil is ingested at night, sufficient drug levels may be present during sleep or the next morning.

The majority of affected users of phosphodiesterase type 5 inhibitors suffer already from other possible risk factors for NAION. It is not possible to determine whether these changes are directly related to sildenafil, sexual activity, the patient's underlying cardiovascular, a combination of these factors, or the other factors [15].

In the present case, the patient had mild hypercholesterolemia without any other vascular predisposing risk factors. However, the family history of his father having suffered from attacks of NAION indicates the possible presence of anatomical or other unidentified risk factors for the development of NAION.

The role of hereditary factors in familial NAION remains unknown, and the only clinical difference between classical and familial NAION is that the familial type seems to have an earlier onset and a higher frequency of bilateral disease [16].

This case might support the association between NAION and the use of erectile dysfunction drugs. Many patients do no disclose to their ophthalmologist information regarding sildenafil intake. Thus, Physician should directly ask about the possible use of this medication and the presence of a family history of NAION among other risk factors before prescribing erectile dysfunction drugs and advising patients to stop the drug if blurry vision occurs. Also, we recommend that patients with a history of monocular NAION be cautioned that sildenafil may increase the risk of NAION in the fellow eye.
